# Crystal structure of 1,7,8,9-tetra­chloro-4-(3,5-di­chloro­benz­yl)-10,10-dimeth­oxy-4-aza­tri­cyclo­[5.2.1.0^2,6^]dec-8-ene-3,5-dione

**DOI:** 10.1107/S2056989014025961

**Published:** 2015-01-01

**Authors:** He Liu, Jia-liang Zhong, Wen-xia Sun, Yan-qing Gong, Li-hong Liu

**Affiliations:** a Beijing Chao-Yang Hospital, Capital Medical University, Beijing 100020, People’s Republic of China; b Shanghai Institute of Pharmaceutical Industry, Shanghai 200040, People’s Republic of China; c State Key Laboratory of Bio-Organic & Natural Products Chmemistry, Shanghai Institute of Organic Chemistry, CAS., Shanghai 200032, People’s Republic of China

**Keywords:** crystal structure, tri­cyclo­[5.2.1.0^2,6^]dec-8-ene-3,5-dione, biological activity, cyclo­alkene skeleton, dipole–dipole inter­actions, hydrogen bonding

## Abstract

In the title compound, C_17_H_11_Cl_6_NO_4_, the configuration of the cyclo­alkene skeleton is *endo,cis*. The benzene ring is twisted by 58.94 (8)° from the attached pyrrolidine ring. Two carbonyl groups play a key role in the crystal packing. A short inter­molecular C⋯O distance of 3.017 (3) Å reveals that one carbonyl group is involved in dipole–dipole inter­actions, which link two adjacent enanti­omers into an inversion dimer. Another carbonyl group provides an acceptor for the weak inter­molecular C—H⋯O hydrogen bonds which link these dimers into layers parallel to (011).

## Related literature   

For related crystal structures, see: Shan *et al.* (2012 ▸); Kossakowski *et al.* (2009 ▸). For the biological activity of related compounds, see: Kossakowski *et al.* (2006 ▸, 2008 ▸); Struga *et al.* (2007 ▸).
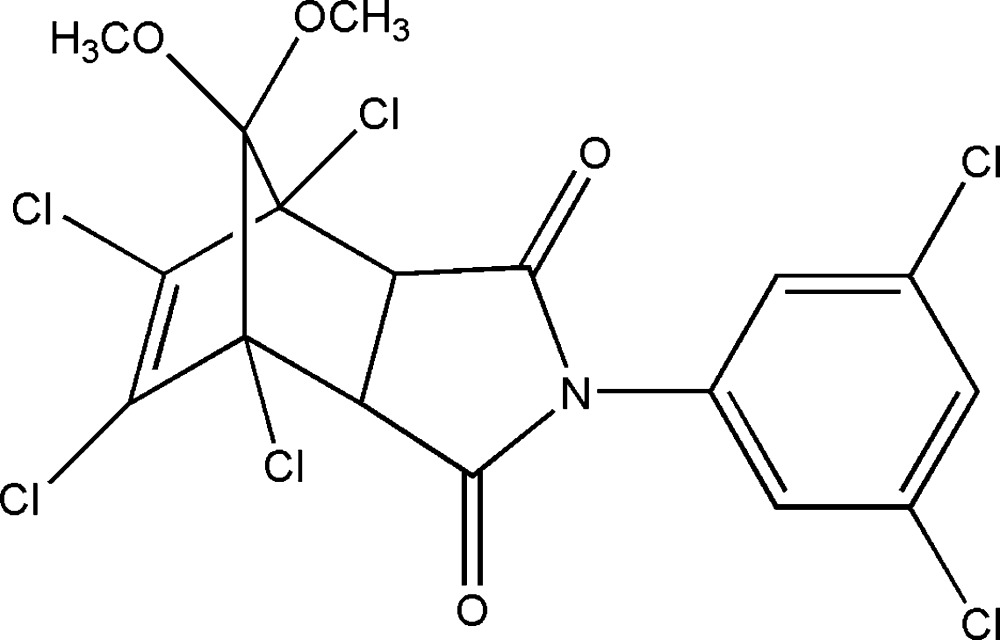


## Experimental   

### Crystal data  

C_17_H_11_Cl_6_NO_4_
*M* *_r_* = 505.97
Triclinic, 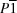

*a* = 8.9905 (18) Å
*b* = 11.351 (2) Å
*c* = 11.482 (2) Å
α = 119.52 (3)°
β = 94.51 (3)°
γ = 90.23 (3)°
*V* = 1015.2 (4) Å^3^
*Z* = 2
Mo *K*α radiation
μ = 0.87 mm^−1^
*T* = 296 K
0.25 × 0.20 × 0.15 mm

### Data collection  

Bruker APEXII CCD diffractometer
10037 measured reflections
4611 independent reflections
3865 reflections with *I* > 2σ(*I*)
*R*_int_ = 0.036

### Refinement  

*R*[*F* ^2^ > 2σ(*F* ^2^)] = 0.043
*wR*(*F* ^2^) = 0.111
*S* = 1.05
4611 reflections
254 parameters
H-atom parameters constrained
Δρ_max_ = 0.70 e Å^−3^
Δρ_min_ = −0.64 e Å^−3^

### 

Data collection: *APEX2* (Bruker, 2009 ▸); cell refinement: *SAINT* (Bruker, 2009 ▸); data reduction: *SAINT*; program(s) used to solve structure: *SHELXS97* (Sheldrick, 2008 ▸); program(s) used to refine structure: *SHELXL97* (Sheldrick, 2008 ▸); molecular graphics: *SHELXTL* (Sheldrick, 2008 ▸); software used to prepare material for publication: *SHELXTL*.

## Supplementary Material

Crystal structure: contains datablock(s) I, global. DOI: 10.1107/S2056989014025961/cv5477sup1.cif

Structure factors: contains datablock(s) I. DOI: 10.1107/S2056989014025961/cv5477Isup2.hkl

Supporting information file. DOI: 10.1107/S2056989014025961/cv5477Isup3.cml

**Fig. 1.**
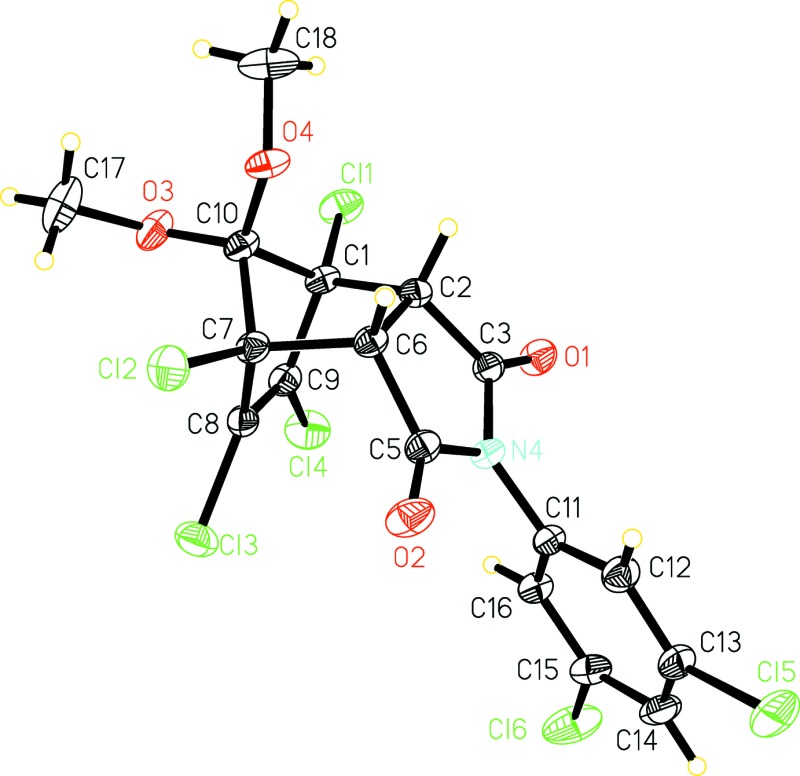
View of (I) showing the atom-labelling scheme. Displacement ellipsoids are drawn at the 30% probability level.

. DOI: 10.1107/S2056989014025961/cv5477fig1.tif
View of (I) showing the atom-labelling scheme. Displacement ellipsoids are drawn at the 30% probability level.

**Fig. 2.**
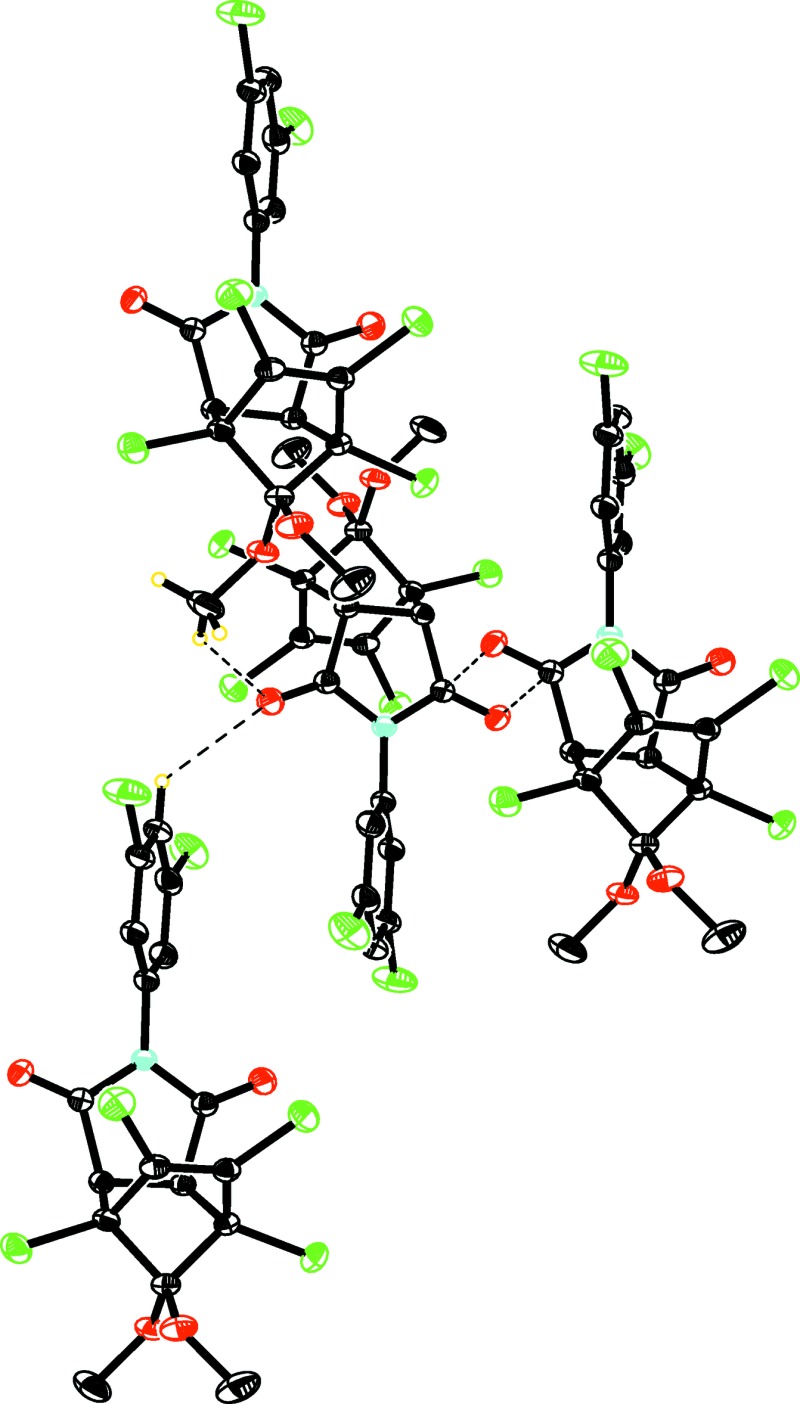
A portion of the crystal packing viewed approximately along [01-1]. The dipole-dipole and intermolecular C—H···O interactions are shown by dashed lines. H atoms not involved in C—H···O interactions are omitted for clarity.

. DOI: 10.1107/S2056989014025961/cv5477fig2.tif
A portion of the crystal packing viewed approximately along [01-1]. The dipole-dipole and inter­molecular C—H⋯O inter­actions are shown by dashed lines. H atoms not involved in C—H⋯O inter­actions are omitted for clarity.

CCDC reference: 1036270

Additional supporting information:  crystallographic information; 3D view; checkCIF report

## Figures and Tables

**Table 1:** Hydrogen-bond geometry (, )

*D*H*A*	*D*H	H*A*	*D* *A*	*D*H*A*
C14H14*A*O2^i^	0.93	2.57	3.265(4)	132
C18H18*A*O2^ii^	0.96	2.45	3.260(4)	141

Symmetry codes: (i) 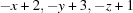; (ii) 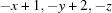.

## supplementary crystallographic information

### S1. Comment

The title compound, (I) (Fig. 1), was synthesized from *N*-(3',5'-dichlorobenzyl)maleimide and 5,5-dimethoxy-1,2,3,4-tetrachlorocydopentadiene. The fused pyrrolidine ring systems are frequently encountered structural units in many synthetically challenging and biologically active alkaloids. The interest of constructing skeletons of this type was further enlightened by the recent disclosure that the rigid arylcyclo analogues having azatricyclo ring systems show anti-HIV-1, anti-cancer, antiviral, and antibacterial activities (Kossakowski *et al.*, 2006).

In (I), the configuration of the cycloalkene skeleton is *endo, cis*. The dihedral angle of pyrrolidine ring and benzene ring is 58.94 (8)°. Two carbonyl groups play a key role in the crystal packing (Fig. 2). One carbonyl group is involved in dipole-dipole interactions, with C3···O1(-*x*+2, -*y*+2, -*z*) distance of 3.017 (3) Å, which link two adjacent enantiomers into inversion dimers. The other carbonyl group provides an acceptor for two weak intermolecular C—H···O hydrogen bonds (Table 1). These intermoleclular interactions link these dimers into layers parallel to (011).

### S2. Experimental

*N*-(3,5-Dichlorobenzyl)maleimide (2.44 g, 10 mmol) and 5,5-dimethoxy-1,2,3,4-tetrachlorocydopentadiene (2.63 g, 10 mmol) were dissolved in anhydrous toluene (100 ml). Then, the solution was refluxed for 8 h. After the solvent was removed under reduced pressure, the residue was dissolved in ether (150 ml), washed with water and brine, dried over anhydrous sodium sulfate, and concentrated to dryness. The product was purified by flash-chromatography (petroleum ether/ethyl acetate, 6:1) and the title compound was isolated as a white solid (4.16 g, 82%), with melting point between 104 and 106°C.

The crystals appropriate for X-ray data collection were obtained from acetone solution at room temperature after four days.

### S3. Refinement

All H atoms were placed in geometically idealized positions and constrained to ride on their parent atoms with C—H distances of 0.93 Å (0.98 for alicylic CH) for aromatic ring CH, and *U*_iso_(H) = 1.2–1.5 *U*_eq_(C).

### Crystal data

**Table:**

C_17_H_11_Cl_6_NO_4_	*Z* = 2
*M**_r_* = 505.97	*F*(000) = 508
Triclinic, *P*1	*D*_x_ = 1.655 Mg m^−^^3^
Hall symbol: -P 1	Mo *K*α radiation, λ = 0.71073 Å
*a* = 8.9905 (18) Å	Cell parameters from 4035 reflections
*b* = 11.351 (2) Å	θ = 3.0–27.3°
*c* = 11.482 (2) Å	µ = 0.87 mm^−^^1^
α = 119.52 (3)°	*T* = 296 K
β = 94.51 (3)°	Column, colourless
γ = 90.23 (3)°	0.25 × 0.20 × 0.15 mm
*V* = 1015.2 (4) Å^3^	

### Data collection

**Table:**

Bruker APEXII CCD diffractometer	3865 reflections with *I* > 2σ(*I*)
Radiation source: fine-focus sealed tube	*R*_int_ = 0.036
Graphite monochromator	θ_max_ = 27.5°, θ_min_ = 3.0°
φ and ω scans	*h* = −10→11
10037 measured reflections	*k* = −14→14
4611 independent reflections	*l* = −14→14

### Refinement

**Table:**

Refinement on *F*^2^	Secondary atom site location: difference Fourier map
Least-squares matrix: full	Hydrogen site location: inferred from neighbouring sites
*R*[*F*^2^ > 2σ(*F*^2^)] = 0.043	H-atom parameters constrained
*wR*(*F*^2^) = 0.111	*w* = 1/[σ^2^(*F*_o_^2^) + (0.0412*P*)^2^ + 0.6429*P*] where *P* = (*F*_o_^2^ + 2*F*_c_^2^)/3
*S* = 1.05	(Δ/σ)_max_ = 0.001
4611 reflections	Δρ_max_ = 0.70 e Å^−^^3^
254 parameters	Δρ_min_ = −0.64 e Å^−^^3^
0 restraints	Extinction correction: *SHELXL97* (Sheldrick, 2008), Fc^*^=kFc[1+0.001xFc^2^λ^3^/sin(2θ)]^-1/4^
Primary atom site location: structure-invariant direct methods	Extinction coefficient: 0.041 (3)

### Special details

**Table:**

Geometry. All e.s.d.'s (except the e.s.d. in the dihedral angle between two l.s. planes) are estimated using the full covariance matrix. The cell e.s.d.'s are taken into account individually in the estimation of e.s.d.'s in distances, angles and torsion angles; correlations between e.s.d.'s in cell parameters are only used when they are defined by crystal symmetry. An approximate (isotropic) treatment of cell e.s.d.'s is used for estimating e.s.d.'s involving l.s. planes.
Refinement. Refinement of *F*^2^ against ALL reflections. The weighted *R*-factor *wR* and goodness of fit *S* are based on *F*^2^, conventional *R*-factors *R* are based on *F*, with *F* set to zero for negative *F*^2^. The threshold expression of *F*^2^ > σ(*F*^2^) is used only for calculating *R*-factors(gt) *etc*. and is not relevant to the choice of reflections for refinement. *R*-factors based on *F*^2^ are statistically about twice as large as those based on *F*, and *R*- factors based on ALL data will be even larger.

### Fractional atomic coordinates and isotropic or equivalent isotropic displacement parameters (Å^2^)

**Table:**

	*x*	*y*	*z*	*U*_iso_*/*U*_eq_	
Cl1	0.86211 (7)	0.64648 (6)	0.01529 (7)	0.04952 (18)	
Cl2	0.42071 (6)	1.01968 (7)	0.22921 (7)	0.04994 (18)	
Cl3	0.68066 (8)	1.06565 (7)	0.47167 (6)	0.05378 (19)	
Cl4	0.95541 (7)	0.84784 (7)	0.33571 (7)	0.05465 (19)	
Cl5	1.13965 (11)	1.59013 (7)	0.35138 (9)	0.0729 (3)	
Cl6	1.36383 (11)	1.31140 (9)	0.58471 (9)	0.0808 (3)	
O1	1.08016 (17)	0.94674 (16)	0.09979 (18)	0.0439 (4)	
O2	0.72222 (19)	1.24926 (16)	0.29413 (19)	0.0498 (4)	
O3	0.54476 (19)	0.70159 (18)	0.11801 (19)	0.0484 (4)	
O4	0.53317 (18)	0.77943 (16)	−0.03513 (15)	0.0412 (4)	
N4	0.92584 (19)	1.11941 (17)	0.21831 (17)	0.0314 (4)	
C1	0.7734 (2)	0.8000 (2)	0.1025 (2)	0.0314 (4)	
C2	0.8098 (2)	0.9081 (2)	0.0613 (2)	0.0298 (4)	
H2A	0.8017	0.8681	−0.0367	0.036*	
C3	0.9573 (2)	0.9867 (2)	0.1245 (2)	0.0314 (4)	
C5	0.7731 (2)	1.1424 (2)	0.2225 (2)	0.0330 (4)	
C6	0.6890 (2)	1.0109 (2)	0.1241 (2)	0.0299 (4)	
H6A	0.6270	1.0203	0.0553	0.036*	
C7	0.5955 (2)	0.9495 (2)	0.1918 (2)	0.0323 (4)	
C8	0.6949 (2)	0.9539 (2)	0.3068 (2)	0.0335 (4)	
C9	0.8003 (2)	0.8677 (2)	0.2542 (2)	0.0343 (5)	
C10	0.5982 (2)	0.7951 (2)	0.0854 (2)	0.0340 (5)	
C11	1.0373 (2)	1.2261 (2)	0.2981 (2)	0.0333 (4)	
C12	1.0362 (3)	1.3406 (2)	0.2843 (2)	0.0409 (5)	
H12A	0.9684	1.3467	0.2222	0.049*	
C13	1.1394 (3)	1.4455 (2)	0.3660 (3)	0.0449 (6)	
C14	1.2412 (3)	1.4386 (2)	0.4583 (3)	0.0472 (6)	
H14A	1.3094	1.5104	0.5128	0.057*	
C15	1.2391 (3)	1.3220 (2)	0.4674 (2)	0.0438 (5)	
C16	1.1374 (2)	1.2143 (2)	0.3883 (2)	0.0371 (5)	
H16A	1.1369	1.1365	0.3960	0.044*	
C17	0.3854 (3)	0.6908 (4)	0.1208 (4)	0.0770 (10)	
H17A	0.3617	0.6226	0.1436	0.115*	
H17B	0.3366	0.6663	0.0339	0.115*	
H17C	0.3516	0.7763	0.1866	0.115*	
C18	0.5309 (4)	0.6462 (3)	−0.1513 (3)	0.0659 (9)	
H18A	0.4837	0.6481	−0.2282	0.099*	
H18B	0.4761	0.5833	−0.1356	0.099*	
H18C	0.6314	0.6183	−0.1673	0.099*	

### Atomic displacement parameters (Å^2^)

**Table:**

	*U*^11^	*U*^22^	*U*^33^	*U*^12^	*U*^13^	*U*^23^
Cl1	0.0530 (4)	0.0289 (3)	0.0557 (4)	0.0074 (2)	−0.0005 (3)	0.0132 (3)
Cl2	0.0286 (3)	0.0601 (4)	0.0557 (4)	0.0069 (2)	0.0054 (3)	0.0243 (3)
Cl3	0.0559 (4)	0.0620 (4)	0.0311 (3)	0.0005 (3)	0.0030 (3)	0.0138 (3)
Cl4	0.0498 (4)	0.0656 (4)	0.0509 (4)	0.0087 (3)	−0.0132 (3)	0.0330 (3)
Cl5	0.1059 (7)	0.0302 (3)	0.0815 (5)	−0.0056 (3)	0.0087 (5)	0.0269 (3)
Cl6	0.0848 (6)	0.0607 (5)	0.0737 (5)	−0.0117 (4)	−0.0449 (4)	0.0228 (4)
O1	0.0299 (8)	0.0397 (9)	0.0530 (10)	0.0028 (6)	0.0050 (7)	0.0157 (8)
O2	0.0422 (9)	0.0303 (8)	0.0605 (11)	0.0061 (7)	0.0023 (8)	0.0103 (8)
O3	0.0439 (9)	0.0454 (10)	0.0604 (11)	−0.0151 (7)	−0.0078 (8)	0.0314 (9)
O4	0.0420 (9)	0.0352 (8)	0.0356 (8)	−0.0046 (6)	−0.0142 (7)	0.0117 (7)
N4	0.0293 (9)	0.0271 (8)	0.0332 (9)	−0.0022 (6)	−0.0018 (7)	0.0121 (7)
C1	0.0332 (10)	0.0242 (9)	0.0327 (10)	−0.0013 (7)	−0.0050 (8)	0.0120 (8)
C2	0.0312 (10)	0.0291 (10)	0.0269 (9)	0.0003 (8)	−0.0012 (8)	0.0127 (8)
C3	0.0327 (11)	0.0302 (10)	0.0304 (10)	−0.0008 (8)	0.0009 (8)	0.0147 (8)
C5	0.0340 (11)	0.0291 (10)	0.0357 (10)	0.0007 (8)	−0.0008 (9)	0.0163 (9)
C6	0.0289 (10)	0.0287 (10)	0.0308 (10)	−0.0007 (7)	−0.0033 (8)	0.0147 (8)
C7	0.0259 (10)	0.0343 (11)	0.0328 (10)	−0.0009 (8)	−0.0019 (8)	0.0143 (9)
C8	0.0329 (11)	0.0382 (11)	0.0295 (10)	−0.0052 (8)	−0.0013 (8)	0.0175 (9)
C9	0.0345 (11)	0.0368 (11)	0.0354 (11)	−0.0046 (8)	−0.0063 (9)	0.0222 (9)
C10	0.0326 (11)	0.0315 (10)	0.0352 (10)	−0.0053 (8)	−0.0068 (9)	0.0159 (9)
C11	0.0337 (11)	0.0279 (10)	0.0329 (10)	−0.0038 (8)	0.0024 (8)	0.0109 (8)
C12	0.0456 (13)	0.0327 (11)	0.0433 (12)	0.0004 (9)	0.0026 (10)	0.0183 (10)
C13	0.0556 (15)	0.0258 (10)	0.0477 (13)	−0.0016 (9)	0.0114 (12)	0.0128 (10)
C14	0.0459 (14)	0.0309 (11)	0.0450 (13)	−0.0068 (9)	0.0004 (11)	0.0041 (10)
C15	0.0429 (13)	0.0364 (12)	0.0381 (11)	−0.0010 (9)	−0.0052 (10)	0.0089 (10)
C16	0.0394 (12)	0.0309 (11)	0.0360 (11)	−0.0017 (8)	−0.0003 (9)	0.0134 (9)
C17	0.0500 (17)	0.078 (2)	0.113 (3)	−0.0235 (16)	0.0000 (18)	0.056 (2)
C18	0.076 (2)	0.0465 (16)	0.0449 (15)	0.0048 (14)	−0.0211 (14)	0.0028 (13)

### Geometric parameters (Å, º)

**Table:**

Cl1—C1	1.758 (2)	C5—C6	1.507 (3)
Cl2—C7	1.752 (2)	C6—C7	1.558 (3)
Cl3—C8	1.698 (2)	C6—H6A	0.9800
Cl4—C9	1.696 (2)	C7—C8	1.514 (3)
Cl5—C13	1.730 (2)	C7—C10	1.568 (3)
Cl6—C15	1.735 (3)	C8—C9	1.318 (3)
O1—C3	1.197 (3)	C11—C16	1.375 (3)
O2—C5	1.199 (3)	C11—C12	1.385 (3)
O3—C10	1.383 (3)	C12—C13	1.383 (3)
O3—C17	1.442 (3)	C12—H12A	0.9300
O4—C10	1.385 (3)	C13—C14	1.377 (4)
O4—C18	1.440 (3)	C14—C15	1.379 (4)
N4—C5	1.399 (3)	C14—H14A	0.9300
N4—C3	1.400 (3)	C15—C16	1.386 (3)
N4—C11	1.436 (3)	C16—H16A	0.9300
C1—C9	1.515 (3)	C17—H17A	0.9600
C1—C2	1.558 (3)	C17—H17B	0.9600
C1—C10	1.569 (3)	C17—H17C	0.9600
C2—C3	1.511 (3)	C18—H18A	0.9600
C2—C6	1.538 (3)	C18—H18B	0.9600
C2—H2A	0.9800	C18—H18C	0.9600
			
C10—O3—C17	117.0 (2)	C8—C9—Cl4	127.64 (18)
C10—O4—C18	117.17 (19)	C1—C9—Cl4	123.58 (17)
C5—N4—C3	113.59 (17)	O3—C10—O4	113.93 (18)
C5—N4—C11	121.92 (17)	O3—C10—C7	118.24 (19)
C3—N4—C11	124.39 (18)	O4—C10—C7	106.81 (17)
C9—C1—C2	107.61 (16)	O3—C10—C1	108.32 (17)
C9—C1—C10	100.14 (17)	O4—C10—C1	116.35 (18)
C2—C1—C10	100.16 (16)	C7—C10—C1	91.53 (15)
C9—C1—Cl1	114.51 (15)	C16—C11—C12	122.2 (2)
C2—C1—Cl1	114.82 (15)	C16—C11—N4	119.76 (19)
C10—C1—Cl1	117.67 (14)	C12—C11—N4	118.0 (2)
C3—C2—C6	105.59 (16)	C13—C12—C11	117.7 (2)
C3—C2—C1	114.53 (17)	C13—C12—H12A	121.2
C6—C2—C1	102.43 (16)	C11—C12—H12A	121.2
C3—C2—H2A	111.3	C14—C13—C12	122.2 (2)
C6—C2—H2A	111.3	C14—C13—Cl5	119.21 (19)
C1—C2—H2A	111.3	C12—C13—Cl5	118.6 (2)
O1—C3—N4	124.78 (19)	C13—C14—C15	118.0 (2)
O1—C3—C2	127.70 (19)	C13—C14—H14A	121.0
N4—C3—C2	107.52 (17)	C15—C14—H14A	121.0
O2—C5—N4	124.4 (2)	C14—C15—C16	122.0 (2)
O2—C5—C6	127.7 (2)	C14—C15—Cl6	119.06 (19)
N4—C5—C6	107.93 (17)	C16—C15—Cl6	118.9 (2)
C5—C6—C2	105.31 (16)	C11—C16—C15	117.9 (2)
C5—C6—C7	113.74 (17)	C11—C16—H16A	121.0
C2—C6—C7	103.90 (16)	C15—C16—H16A	121.0
C5—C6—H6A	111.2	O3—C17—H17A	109.5
C2—C6—H6A	111.2	O3—C17—H17B	109.5
C7—C6—H6A	111.2	H17A—C17—H17B	109.5
C8—C7—C6	107.50 (16)	O3—C17—H17C	109.5
C8—C7—C10	100.17 (17)	H17A—C17—H17C	109.5
C6—C7—C10	99.80 (16)	H17B—C17—H17C	109.5
C8—C7—Cl2	116.77 (15)	O4—C18—H18A	109.5
C6—C7—Cl2	112.98 (15)	O4—C18—H18B	109.5
C10—C7—Cl2	117.57 (15)	H18A—C18—H18B	109.5
C9—C8—C7	107.49 (18)	O4—C18—H18C	109.5
C9—C8—Cl3	127.66 (18)	H18A—C18—H18C	109.5
C7—C8—Cl3	124.45 (16)	H18B—C18—H18C	109.5
C8—C9—C1	108.25 (19)		
			
C9—C1—C2—C3	48.4 (2)	Cl1—C1—C9—C8	−160.38 (16)
C10—C1—C2—C3	152.57 (17)	C2—C1—C9—Cl4	−101.47 (19)
Cl1—C1—C2—C3	−80.38 (19)	C10—C1—C9—Cl4	154.36 (16)
C9—C1—C2—C6	−65.4 (2)	Cl1—C1—C9—Cl4	27.5 (2)
C10—C1—C2—C6	38.79 (18)	C17—O3—C10—O4	−54.4 (3)
Cl1—C1—C2—C6	165.84 (13)	C17—O3—C10—C7	72.3 (3)
C5—N4—C3—O1	176.3 (2)	C17—O3—C10—C1	174.4 (2)
C11—N4—C3—O1	−0.1 (3)	C18—O4—C10—O3	−51.2 (3)
C5—N4—C3—C2	−2.6 (2)	C18—O4—C10—C7	176.3 (2)
C11—N4—C3—C2	−178.97 (18)	C18—O4—C10—C1	75.9 (3)
C6—C2—C3—O1	−176.5 (2)	C8—C7—C10—O3	60.5 (2)
C1—C2—C3—O1	71.6 (3)	C6—C7—C10—O3	170.41 (18)
C6—C2—C3—N4	2.3 (2)	Cl2—C7—C10—O3	−67.1 (2)
C1—C2—C3—N4	−109.57 (19)	C8—C7—C10—O4	−169.50 (16)
C3—N4—C5—O2	−178.8 (2)	C6—C7—C10—O4	−59.55 (19)
C11—N4—C5—O2	−2.4 (3)	Cl2—C7—C10—O4	62.9 (2)
C3—N4—C5—C6	1.8 (2)	C8—C7—C10—C1	−51.33 (17)
C11—N4—C5—C6	178.21 (18)	C6—C7—C10—C1	58.61 (17)
O2—C5—C6—C2	−179.5 (2)	Cl2—C7—C10—C1	−178.91 (15)
N4—C5—C6—C2	−0.1 (2)	C9—C1—C10—O3	−70.1 (2)
O2—C5—C6—C7	−66.4 (3)	C2—C1—C10—O3	179.76 (16)
N4—C5—C6—C7	112.96 (19)	Cl1—C1—C10—O3	54.6 (2)
C3—C2—C6—C5	−1.3 (2)	C9—C1—C10—O4	160.04 (18)
C1—C2—C6—C5	118.88 (17)	C2—C1—C10—O4	49.9 (2)
C3—C2—C6—C7	−121.17 (17)	Cl1—C1—C10—O4	−75.2 (2)
C1—C2—C6—C7	−0.97 (19)	C9—C1—C10—C7	50.40 (18)
C5—C6—C7—C8	−47.1 (2)	C2—C1—C10—C7	−59.74 (17)
C2—C6—C7—C8	66.9 (2)	Cl1—C1—C10—C7	175.13 (15)
C5—C6—C7—C10	−151.10 (17)	C5—N4—C11—C16	121.4 (2)
C2—C6—C7—C10	−37.14 (18)	C3—N4—C11—C16	−62.5 (3)
C5—C6—C7—Cl2	83.22 (19)	C5—N4—C11—C12	−56.3 (3)
C2—C6—C7—Cl2	−162.83 (13)	C3—N4—C11—C12	119.8 (2)
C6—C7—C8—C9	−68.0 (2)	C16—C11—C12—C13	−0.6 (3)
C10—C7—C8—C9	35.8 (2)	N4—C11—C12—C13	177.0 (2)
Cl2—C7—C8—C9	163.88 (16)	C11—C12—C13—C14	0.2 (4)
C6—C7—C8—Cl3	105.23 (19)	C11—C12—C13—Cl5	−179.64 (17)
C10—C7—C8—Cl3	−151.01 (16)	C12—C13—C14—C15	0.4 (4)
Cl2—C7—C8—Cl3	−22.9 (2)	Cl5—C13—C14—C15	−179.70 (19)
C7—C8—C9—C1	−1.3 (2)	C13—C14—C15—C16	−0.8 (4)
Cl3—C8—C9—C1	−174.27 (16)	C13—C14—C15—Cl6	−178.90 (19)
C7—C8—C9—Cl4	170.36 (16)	C12—C11—C16—C15	0.3 (3)
Cl3—C8—C9—Cl4	−2.6 (3)	N4—C11—C16—C15	−177.3 (2)
C2—C1—C9—C8	70.7 (2)	C14—C15—C16—C11	0.4 (4)
C10—C1—C9—C8	−33.5 (2)	Cl6—C15—C16—C11	178.55 (17)

### Hydrogen-bond geometry (Å, º)

**Table:**

*D*—H···*A*	*D*—H	H···*A*	*D*···*A*	*D*—H···*A*
C14—H14*A*···O2^i^	0.93	2.57	3.265 (4)	132
C18—H18*A*···O2^ii^	0.96	2.45	3.260 (4)	141

Symmetry codes: (i) −*x*+2, −*y*+3, −*z*+1; (ii) −*x*+1, −*y*+2, −*z*.
