# Supplementary figures and images for: Crystal structure of 1,7,8,9-tetra­chloro-4-(3,5-di­chloro­benz­yl)-10,10-dimeth­oxy-4-aza­tri­cyclo­[5.2.1.02,6]dec-8-ene-3,5-dione
Source: Acta Crystallogr E Crystallogr Commun. 2015 Jan 1;71(Pt 1):o14–5. doi: 10.1107/S2056989014025961 (PMC4331899; doi:10.1107/S2056989014025961)

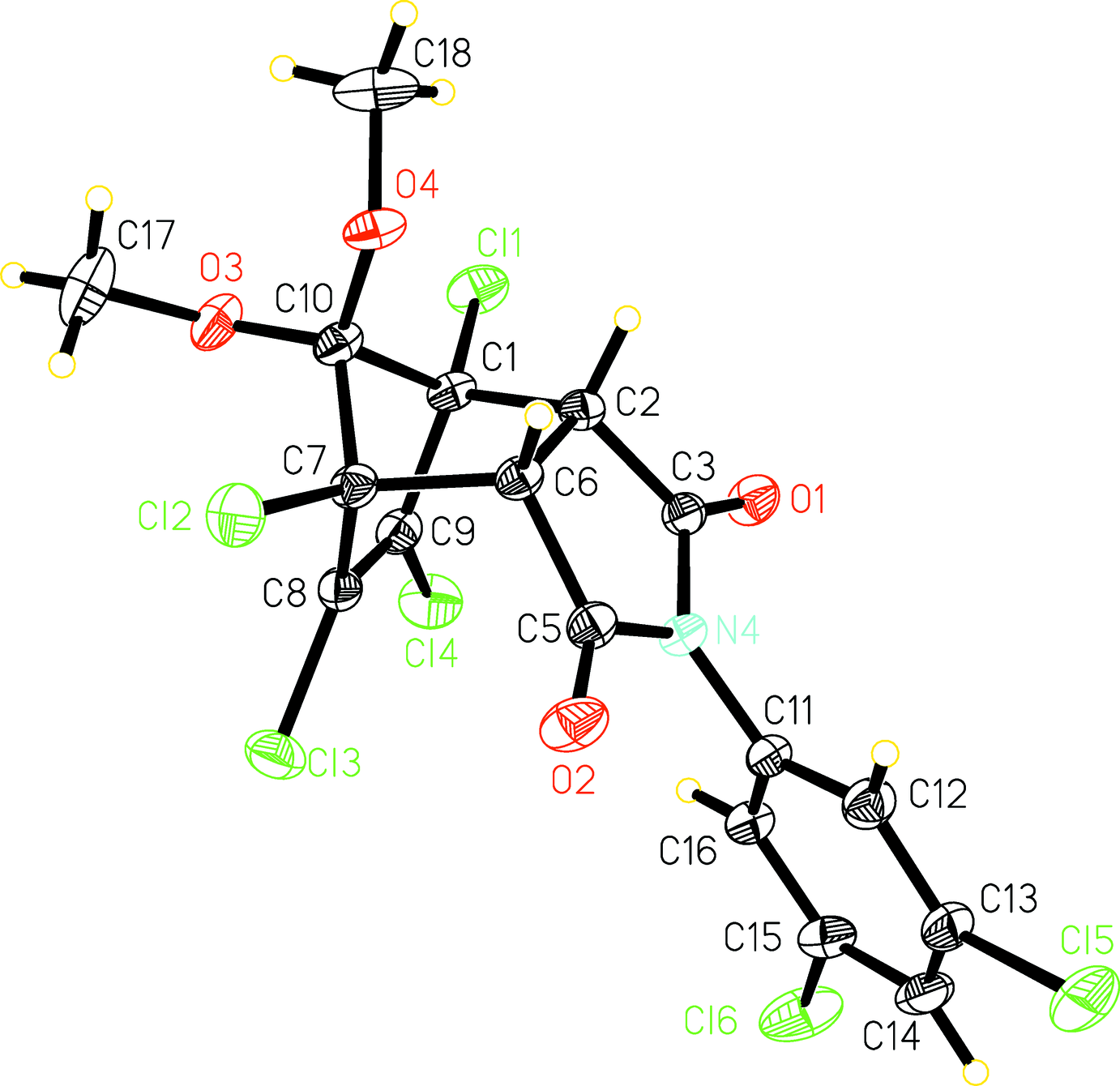

Supplement: Supplementary file 4 [file e-71-00o14-fig1.tif]

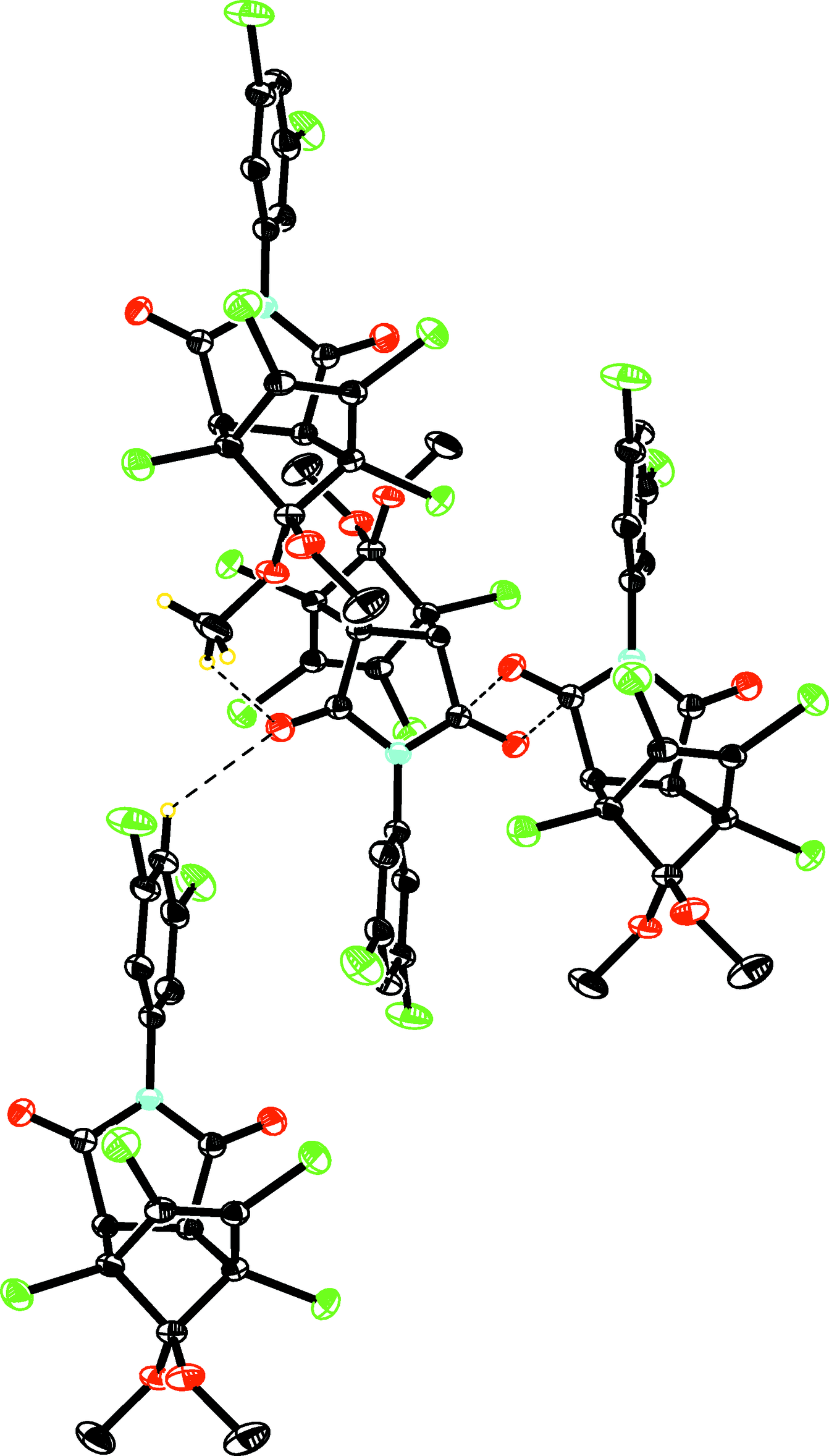

Supplement: Supplementary file 5 [file e-71-00o14-fig2.tif]
